# Endogenous controls in human umbilical vein endothelial cells under metabolic and oxidative stress

**DOI:** 10.1186/1471-2164-15-S2-P23

**Published:** 2014-04-02

**Authors:** Sherin Bakhashab, Sahira Lari, Farid Ahmed, Hans-Juergen Schulten, Manikandan Jayapal, Sajjad Karim, Ayat Bashir, Fahad Ahmed, Abdulrahman Al-Malki, Hasan S Jamal, Mamdooh Gari, Mohammed H Alqahtani, Ioakim Spyridopoulos, Jolanta U Weaver

**Affiliations:** 1 Institute of Cellular Medicine, Newcastle University, Newcastle, UK; 2 Biochemistry Department, King Abdulaziz University, Jeddah, KSA; 3 Centre of Excellence in Genomic Medicine Research, King Abdulaziz University, Jeddah, KSA; 4 Faculty of Science, The University of Western Australia, Crawley, Australia; 5 Department of Obstetrics & Gynecology Faculty of Medicine King Abdulaziz University, Jeddah, KSA

## Background

Gene expression studies on the effect of hypoxia and hyperglycemia using human umbilical vein endothelial cell (HUVEC) cultures are of particular interest in cardiovascular disease and diabetes. Normalization of gene expression data refers to the comparison of expression values using endogenous control that is steady across independent experimental conditions, a crucial step for gene expression studies. The endogenous controls for experiments involving hyperglycemia, hypoxia and a combination of the two have not been identified before in HUVEC. Our objective was to identify endogenous controls that are stable under oxidative (hypoxia) and metabolic stress (hyperglycemia) in HUVEC.

## Materials and methods

We applied human genome-wide expression array using Affymetrix^®^ GeneChip^®^ on mRNA obtained from 3 different primary HUVEC cultures incubated in euglycemic (5.5 mM) or hyperglycemic conditions (16.5 mM) and/or in chemical hypoxia induced by 150 µM Cobalt Chloride for 1, 3, 12 hours.

## Results

Microarray data showed that 9560 genes were identified as potential endogenous controls under hypoxia, hyperglycemia, and hyperglycemia combined with hypoxia. Subsequently, the RNA expression level of 5 endogenous controls was validated by real-time quantitative PCR (qRT-PCR) to confirm the stability of the expression. The following endogenous controls were identified as the most stable: under hyperglycemia ribosomal protein, large, P0 (RPLP0), and transferrin receptor (TFRC), Glyceraldehyde-3-phosphate dehydrogenase (GAPDH), glucuronidase, beta (GUSB), and β-actin, under hypoxia alone RPLP0, and TFRC whereas under hyperglycemia combined with hypoxia RPLP0, TFRC, GUSB, and β-actin.

## Conclusions

Our data demonstrate that RPLP0 and TFRC are the most suitable endogenous controls analyzed for expression studies utilizing HUVEC cultured under metabolic or oxidative stress at 1h, 3h, and 12h time points. The other genes were detected to be stable at the short-term but not after long-term exposure to hypoxia.
